# Egg antigen p40 of *Schistosoma japonicum* promotes senescence in activated hepatic stellate cells via SKP2/P27 signaling pathway

**DOI:** 10.1038/s41598-017-00326-1

**Published:** 2017-03-21

**Authors:** Tianhua Xu, Jinling Chen, Dandan Zhu, Liuting Chen, Jianxin Wang, Xiaolei Sun, Bin Hu, Yinong Duan

**Affiliations:** 1 Department of Pathogen Biology, School of Medicine, Nantong University, Nantong, 226001 Jiangsu People’s Republic of China; 2 Department of Cardiology, The First Affiliated Hospital of Nanjing Medical University, Nanjing, 210029 Jiangsu People’s Republic of China; 3 Laboratory Medicine Center, Affiliated Hospital of Nantong University, Nantong, 226001 Jiangsu People’s Republic of China

## Abstract

Schistosomiasis is characterized by egg deposition, granulomatous inflammatory reaction and then subsequent hepatic fibrosis formation. Activated HSCs are regarded as the main effector cells in the progression of liver fibrosis and induction of senescence in hepatic stellate cells (HSCs) is vital to the reversion of hepatic fibrosis. Our previous work has showed that *S*. *japonicum* egg antigen p40 (Sjp40) could promote HSCs senescence via a STAT3/p53/p21 mechanism. In this paper, the major aim was to explore whether there are other signaling pathways in the process of Sjp40-induced HSCs aging and the underlying effect of SKP2/P27 signal pathway in this procedure. We observed the Sjp40-induced decrease of α-SMA and the senescence of LX-2 cells, and Sjp40 could upregulate P27 and downregulate the protein level of SKP2. The senescence induced by Sjp40 might be reversed in LX-2 cells that treated with P27-specific siRNA or with SKP2-special over-expression plasmid. In addition, we also demonstrated that the decreased expression of P-Rb and α-SMA induced by Sjp40 were partly restored by SKP2-overexpression. These data suggest that Sjp40 might inhibit HSCs activation by promoting cellular senescence via SKP2/P27 signaling pathway, which put forward novel mechanism in the treatment of liver fibrosis.

## Introduction

Liver fibrosis, which ultimately could lead to cirrhosis, liver failure, and portal hypertension in advanced hepatic fibrosis, is characterized by the excess deposition of extracellular matrix (ECM) components^1, 2^. Activated hepatic stellate cells (HSCs) is a major source of ECM and a key mediator in liver fibrogenesis. In the process of liver fibrogenesis, quiescent HSCs could transform into activated HSCs, a myofibroblast phenotype, leading to the production of a great amount of ECM and secretion of many kinds of pro-inflammatory and pro-fibrogenic cytokines^3, 4^. Thus, inhibiting HSCs activation and reducing the number of activated HSCs are effective strategies against liver fibrosis^5, 6^.

Schistosomiasis is one of the most important causes of liver fibrosis, which is characterized by egg deposition, granulomatous inflammatory reaction and then subsequent hepatic fibrosis formation^7, 8^. Many researchers have demonstrated the anti-fibrotic effect of schistosoma eggs and soluble egg antigens (SEA). And many studies found that both *Schistosoma mansoni* (*S*. *mansoni*) eggs and *Schistosoma japonicum* (*S*. *japonicum*) eggs could restrain the activation of hepatic stellate cell and induce the down-regulation of fibrogenesis^9, 10^. Previously, we have demonstrated that SEA from *S*. *japonicum* could induce the suppression of activated human HSCs cell lines (LX-2) and primary mice HSCs through the TGFβ and PPARγ signaling pathways^11^. SEA-treated LX-2 cells exhibited cell senescence, cell cycle arrest and cell growth inhibition^12^, and generated cell apoptosis phenomena in caspase-^11^ and p53/DR5-dependent signaling pathway^13^.

SEA is a very complex mixture which is composed of various egg antigens, and some laboratories have isolated multiple antigens from this rough soluble egg antigens, including Smp40 (*S*. *mansoni* egg antigen p40), Sjp40 (*S*. *japonicum* egg antigen p40). It has been reported that Sjp40 has been demonstrated to be a potential antigen used for the early schistosomiasis diagnosis and may be a promising target for prevention and control of the disease^14^. In addition, Abouel-Nour MF *et al*. have also found that IL-10 was obviously increased, whereas IL-5 was significantly reduced in Smp40-treated peripheral blood mononuclear cells from patients infected with *S*. *mansoni* ^15^. Of note, some studies have demonstrated that IL-10 could regress liver fibrosis via suppressing expression of matrix metalloproteinase and collagen^16, 17^, while IL-5 promoted the progression of hepatic fibrosis by the regulation of IL-13 activity^18^. The above evidences seemingly support that Sjp40 might modulate liver fibrosis and exert an anti-fibrosis effect. We have expressed and purified Sjp40, which has been used for stimulation of LX-2 cells *in vitro*. Results have confirmed that Sjp40 inhibits TGF-β1-induced activation of HSCs *in vitro* ^19^.

It is reported that the inhibitory effect of HSCs activation might be accompanied by cell senescence^20^. In our previous study, we have demonstrated that Sjp40 could promote HSCs senescence via a STAT3/p53/p21 dependent mechanism^21^. But this signal pathway interference could not completely rescue the Sjp40-induced senescence. Hence, we speculated that there are other signaling pathways which might be also involved in the process of Sjp40-induced HSCs aging. SKP2/P27 pathway is also known to be one of important senescent signal channels except p53 or p16 pathway, and our experiment showed Sjp40 could induce the expression change of SKP2 and P27. Therefore, in this present paper we attempted to explore the role of SKP2/P27 pathway in Sjp40-induced HSCs senescence and elucidate the underlying molecular mechanism.

## Results

### Inhibitory effect of Sjp40 on HSCs activation is independent on the cell apoptosis

**Figure 1:**
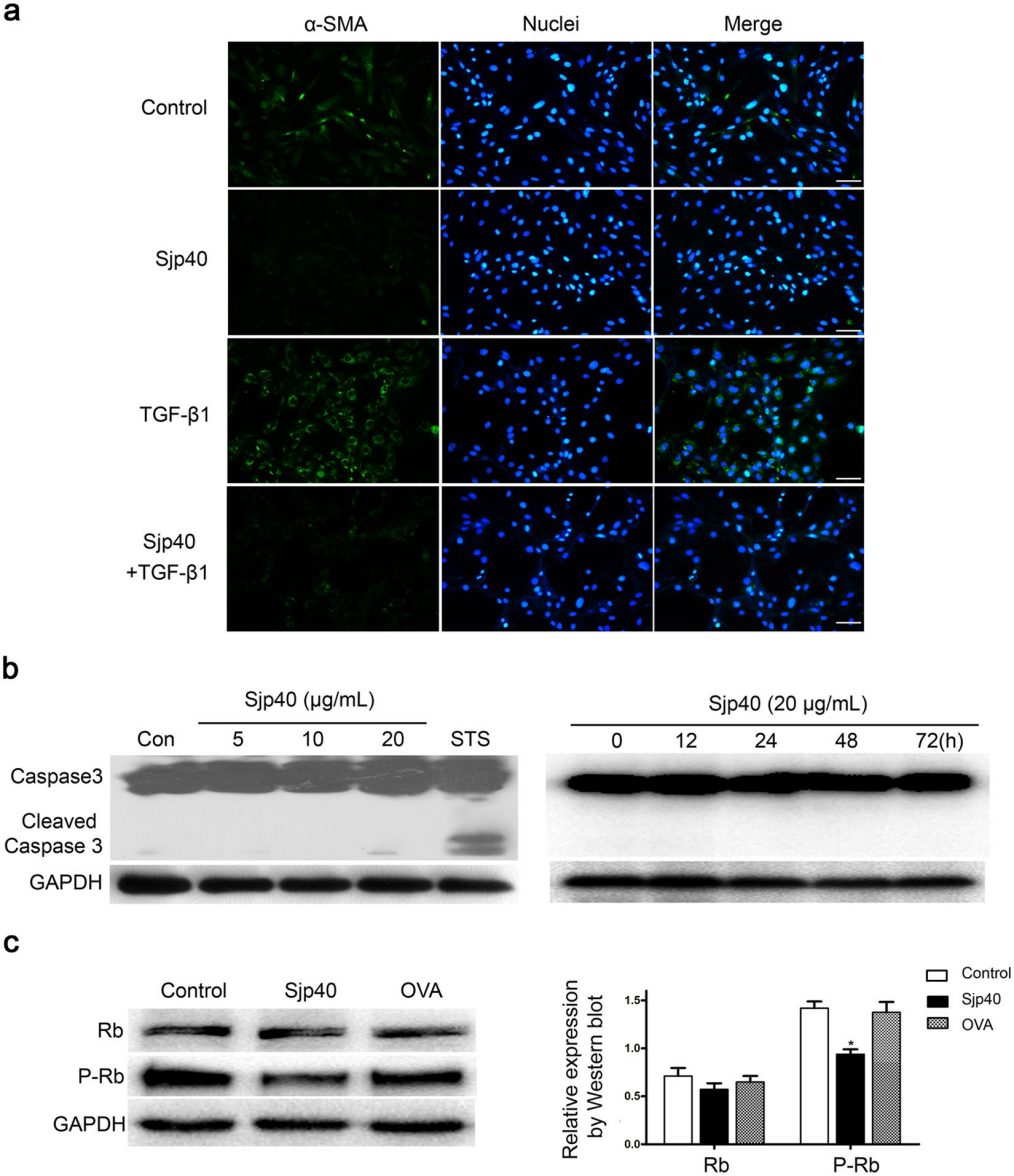
Inhibitory effect of Sjp40 on HSCs activation of LX-2 is independent on the cell apoptosis. (**a**) Effect of Sjp40 on the level of α-SMA in LX-2 cells analyzed by immunofluorescence assay (100× magnification). After treatment with Sjp40 for 48 h, the protein level of α-SMA was measured by immunofluorescence, and Hoechst 33342 was used to stain the nucleus. The cells were photographed using fluorescence microscope. (**b**) Impact of Sjp40 on LX-2 cells apoptosis. The cells were exposed to different concentrations of Sjp40 (5, 10, 20 μg/mL) or Staurosporin (STS) as a positive control, and at the different times (0, 12, 24, 48, 72 h). And then the level of cleaved-caspase-3 was analyzed by Western blot assay. (**c**) Effects of Sjp40 on the expression of cell cycle regulatory proteins. The protein levels of Rb and P-Rb in LX-2 cells treated with or without Sjp40 were analyzed by Western blot assay. *p < 0.05 compared to control group. Bar: 50 micrometers.

It is well known that the activation of HSCs plays a key role in liver fibrogenesis, and activated HSCs are the main source of ECM and characterized by the expression of α-SMA^2^. Previously, we found Sjp40 inhibited the expression of α-SMA assayed by Western blot. Consistent with the results, Sjp40 could also obviously suppress the TGF-β1-induced activation of LX-2 cells, accompanied with down-regulation of α-SMA (Fig. 1a). It is reported that the inhibitory effect of HSCs activation might be accompanied by cell apoptosis^13^ or senescence^22^. Hence, we speculated that cell apoptosis might be involved in the process of Sjp40-induced HSCs inactivation. To prove this hypothesis, the level of cleaved-caspase-3 was applied to investigate the ability of Sjp40 to induce apoptosis in LX-2 cells. As illustrated in Fig. 1b, Sjp40 at different concentration could not induce the expression of cleaved-caspase-3 in LX-2 cells compared with positive group. To further confirm it, the expression of cleaved caspase 3 was measured at the different times (0, 12, 24, 48, 72 h). No significant difference was observed (Fig. 1b), suggesting that inhibitory effect of Sjp40 on HSCs activation is independent on the cell apoptosis.

In our previous studies, Sjp40 could induce cell cycle arrest of LX-2 cells in the G1 phase and corresponding decrease of the number of LX-2 cells at S and G2 phase^21^. To further confirm the role of Sjp40 on cellular senescence, phosphorylation of Rb, which has been viewed as the crucial step in the progression of G1-S phase transition, was measured by Western blot. We observed obvious dephosphorylation of Rb in Sjp40-treated cells (Fig. 1c). Taken together, these results demonstrate that Sjp40-induced inactivation of HSCs is not due to cell apoptosis.

### Cellular senescence induced by Sjp40 is associated with P27 signal pathway

**Figure 2:**
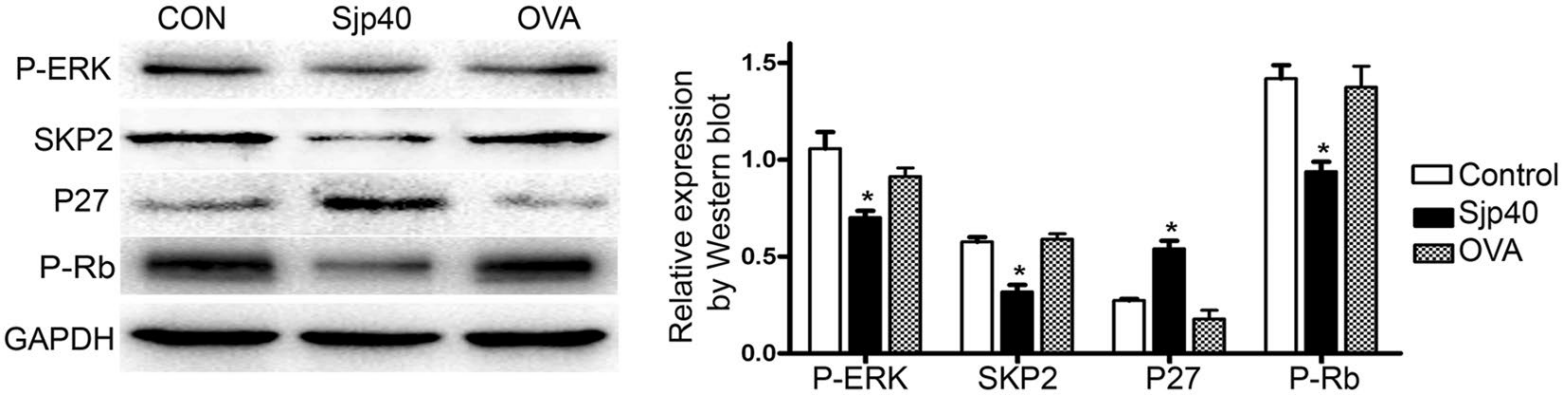
Cellular senescence triggered by Sjp40 is associated with P27 signal pathway. Western blot analysis of the expression levels of P-ERK, SKP2, P27 and P-Rb in LX-2 cells treated with Sjp40. All values were expressed as the mean ± SEM of three or more independent trials. *p < 0.05 compared to control group.

G1-S phase transition has recently been recognized to be mediated by the induction of P27 in ERK-inhibited fibroblasts^23^. P27 signaling pathway is served as an important aging-associated signaling pathway^24^. To prove whether the underlying molecular mechanism of Sjp40-induced senescence and cell cycle arrest is associated with P27-related signal pathways, total proteins extracted from LX-2 cells treated with or without Sjp40 were analyzed by Western blot assay with the specific antibodies against P27, P-ERK, SKP2 and P-Rb. As illustrated in Fig. 2, P-ERK, SKP2 as well as P-Rb in LX-2 cells significantly decreased upon being stimulated by the Sjp40. In contrast, obvious upregulation of P27 was observed in the Sjp40-treated group, compared with control group. Our data revealed that SKP2/P27 signal pathway might be implicated in Sjp40-induced cellular senescence (Fig. 2).

### Cellular senescence induced with Sjp40 is dependent on P27

**Figure 3:**
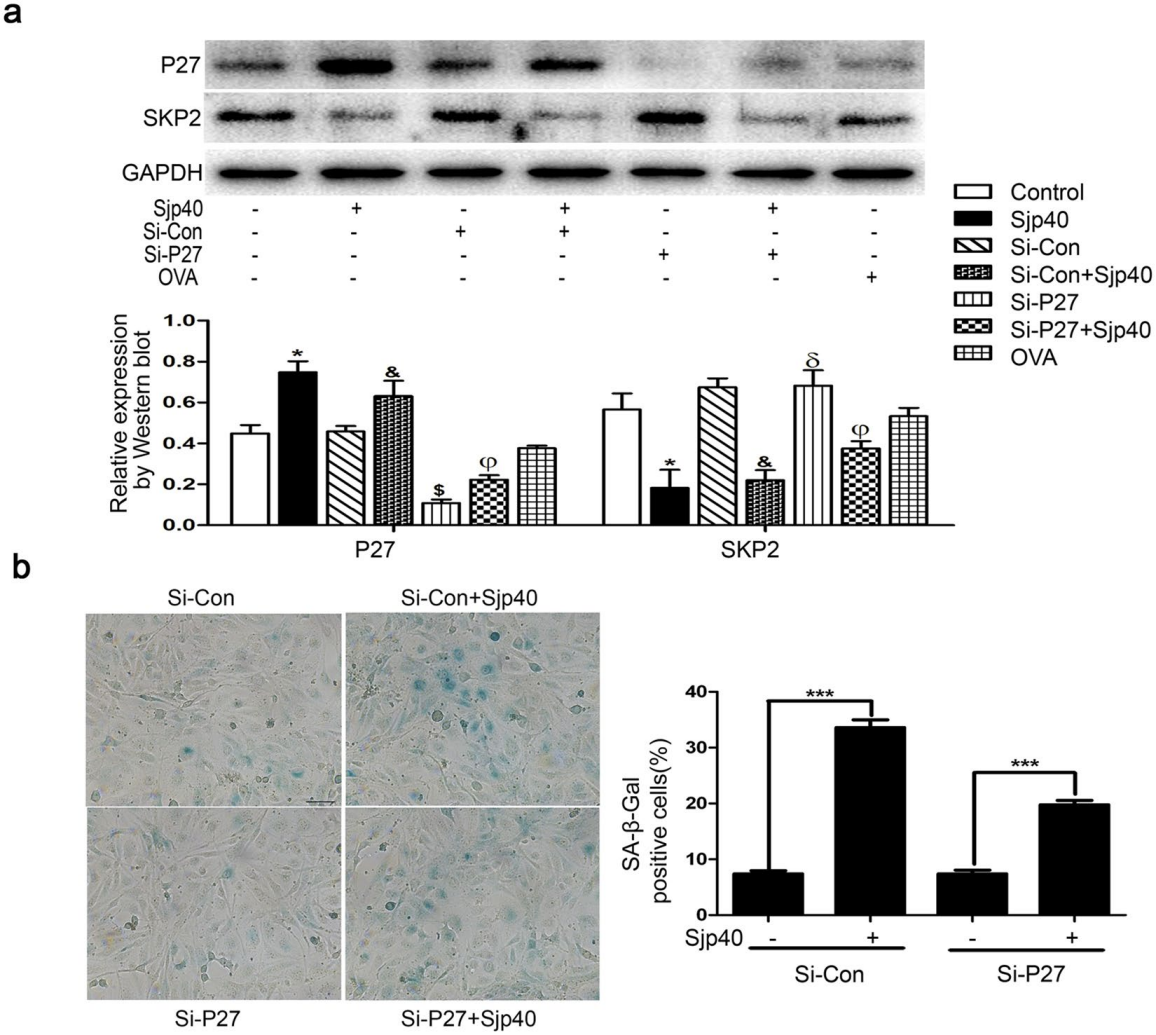
Cellular senescence induced by Sjp40 is dependent on P27. (**a**) LX-2 cells were transfected with Si-P27 or Si-Con and additionally treated with or without Sjp40 for 48 h. The protein levels of SKP2 and P27 were assayed by Western blot and date were expressed as the mean ± SEM of three or more independent trials. *p < 0.05 compared to the control group; ^&^ *p* < 0.05 compared to the Si-con group; ^$^ *p* < 0.05 compared to the control group; ^φ^ *p* < 0.05 compared to Si-P27 group. ^δ^ *p* > 0.05 compared to the Si-con group. (**b**) Knockdown of P27 rescued the Sjp40-induced senescence measured by SA-β-Gal assay (original magnification 200×) and quantitative analysis of the percentage of SA-β-Gal positive cells (%) was expressed as the mean ± SEM of three or more independent trials. ***p < 0.001. Bar: 50 micrometers.

Since Sjp40 treatment markedly increased the protein level of P27, we postulated if P27 silencing might reverse the senescence-like phenotype triggered by Sjp40. LX-2 cells were transiently transfected with P27 Si-RNA or Scrambled Si-RNA. Figure 3a showed that no obvious difference of SKP2 was observed after knockdown of P27. Then, the Sjp40-induced senescence of LX-2 following P27 silencing was analyzed by SA-β-Gal assay. Figure 3b demonstrated that SA-β-Gal positive percent mediated by Sjp40 in LX-2 cells obviously reduced via knockdown of P27, which confirmed the crucial role of P27 in regulating cellular aging induced by Sjp40. In short, P27 is a key mediator in Sjp40-induced cellular senescence.

### Enhanced expression of SKP2 by high expression plasmid restores Sjp40-induced senescence in LX-2 cells

**Figure 4:**
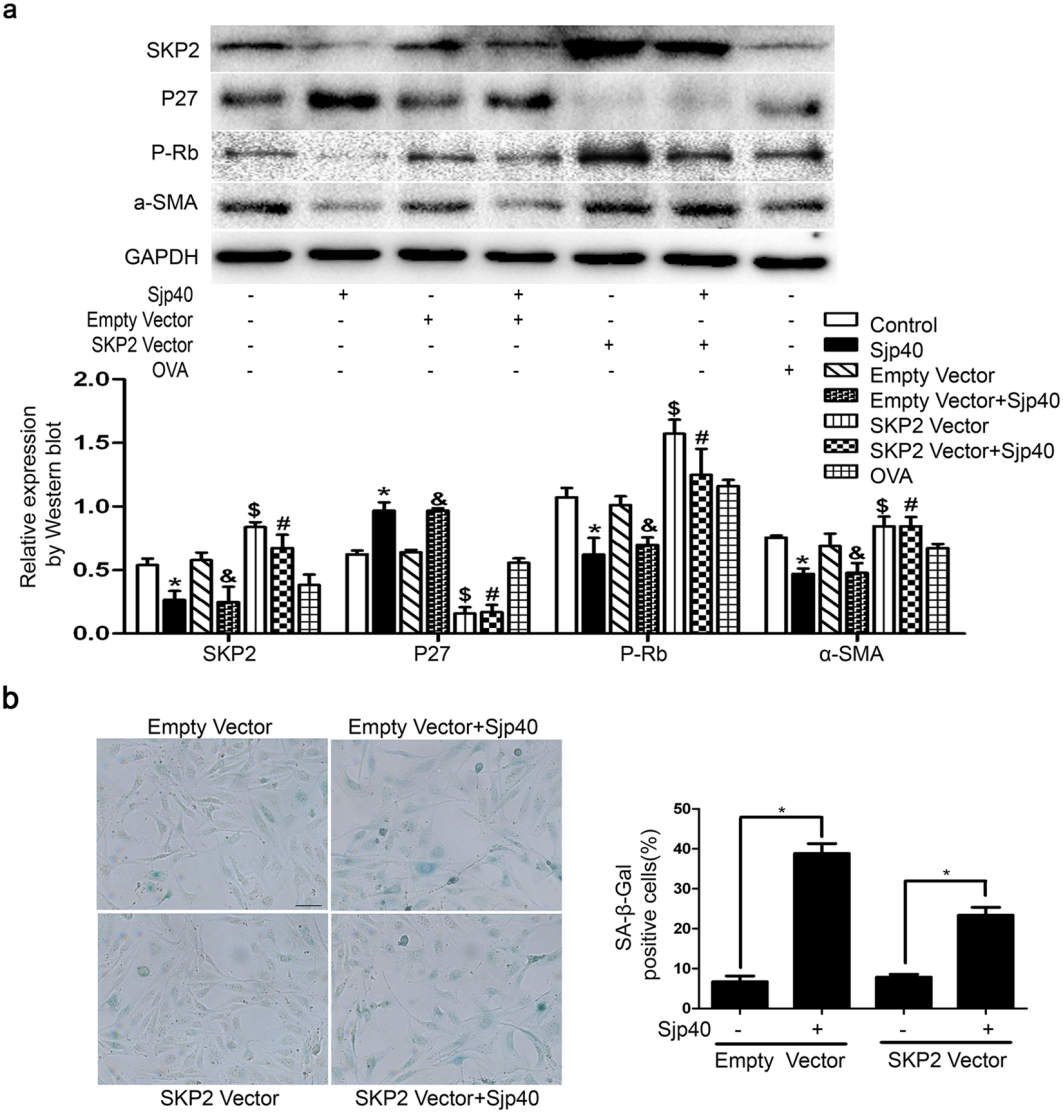
Enhanced expression of SKP2 by high expression plasmid restores Sjp40-induced senescence in LX-2 cells. (**a**) LX-2 cells were transfected with pcDNA3.1 or pcDNA-SKP2 and additionally treated with or without Sjp40 for 48 h. The protein levels of SKP2, P27, P-Rb and α-SMA were investigated by Western blot assay and date were expressed as the mean ± SEM of three or more independent trials. *p < 0.05 compared to the control group; ^&^ *p* < 0.05 compared to the pcDNA3.1 group. ^$^ *p* < 0.05 compared to the control group; ^#^ *p* > 0.05 compared to SKP2-treated group. (**b**) Over-expression of SKP2 rescued the Sjp40-induced senescence analyzed by SA-β-Gal assay (original magnification 200×) and the graph also reveals quantitative analysis of the percentage of SA-β-Gal positive cells (%) expressed as the mean ± SEM of three or more independent trials. *p < 0.05 compared to the control group. Bar: 50 micrometers.

In order to demonstrate whether the positive regulation of P27 expression triggered by Sjp40 is in a SKP2-dependent manner, the recombinant vector pcDNA3.1-SKP2 was constructed and verified by restriction analysis and sequencing (Fig. S1). Then, we forced the expression of SKP2 by transiently transfecting high expression plasmid in LX-2 cells. Figure 4a showed that SKP2 over-expression reversed P27 protein level induced by Sjp40, indicating that P27 expression induced by Sjp40 is dependent on SKP2 in LX-2 cells. In addition, over-expression of SKP2 restored the inhibition of α-SMA and P-Rb expression in Sjp40-treated LX-2 cells (Fig. 4a), demonstrating that SKP2 over-expression might rescue cell inactivation and cell cycle arrest in Sjp40-treated LX-2 cells. In order to further confirm the role of SKP2 in Sjp40-induced senescence, the Sjp40-induced senescence of LX-2 cells following SKP2 over-expression was analyzed by SA-β-Gal assay. Figure 4b demonstrated that enhanced expression of SKP2 reduced the number of SA-β-Gal positive cells being exposed to Sjp40 and partially rescued the Sjp40-induced senescence in LX-2 cells. In a word, SKP2 is involved in Sjp40-induced senescence via regulating P27 protein level in LX-2 cells.

## Discussion

Liver fibrosis, as a common forerunner of many chronic liver diseases, is characterized by excessive deposition of ECM components. The mainstream idea reported in the academic journals until recently was that hepatic fibrosis is irreversible, but more and more researches indicate that fibrosis could be suppressed and potentially even reversed^25^. Liver fibrosis regression usually contains (1) blocking the collagen fibril formation^26^, (2) stopping epithelial cells transforming into myofibroblast (MFB) by preventing epithelial-mesenchymal transition (EMT) process^27^ and (3) activating the killing activity of NK cells to HSCs^6^. These will eventually alleviate hepatic fibrosis, but above all, the suppression of HSCs activity and reduction of the number in activated HSCs are a special focus. Activated HSCs, the main effector cell in the progression of liver fibrosis, is a major source of ECM and can secret large amounts of pro-inflammatory and pro-fibrogenic cytokines^1^, thus, the inhibition and removal of activated HSCs are critical strategies for liver fibrosis treatment, which can be realized by the induction of cellular senescence^22^, triggering cell apoptosis^5^, and enhancing the clearance activity of immunocytes on HSCs^28^.

Cellular senescence refers to an irreversible cell cycle arrest state that could limit cell proliferation and growth^23^. It is generally known that cell senescence is regarded as an effective method of tumor suppression^29^, however, recent researches demonstrate that it also plays an important suppressive role in other non-neoplastic diseases, such as liver fibrosis. The induction of aging in activated HSCs eventually might limit hepatic fibrosis^30^, in contrast, the restriction of the senescence program could alleviate liver fibrogenesis^22^. Once HSCs transformed into MFB by EMT process, it possessed the potent proliferation activity and produced a great deal of ECM leading to promote fibrosis formation. Nevertheless, the senescence could restrain activated-HSCs growth and reduce cellular population, which contribute to reducing ECM secretion, and hopefully realize the reversion of liver fibrosis^31^. In addition, the secretory characteristics will change in senescent activated-HSCs compared to the growing HSCs. The production of α-SMA, TGF-β1, fibronectin, collagen type I and IV will be down-regulated, meanwhile, the expression of matrix metalloproteinases (MMP) effectively degrading ECM, such as MMP-1 and MMP-3, might be up-regulated, which are beneficial to inhibit hepatic fibrosis procedure^22^. Senescent activated-HSCs also up-regulate the level of immuno-regulatory molecules that could activate NK cell function and enhance the killing activity of NK cells to HSCs, eventually contributing to the alleviation of fibrosis^22^.

The p53/p21/P-Rb and p16/CDK4/P-Rb pathways have been recognized as the key signal transduction pathways for regulating cellular aging^32^. In our previous study, we have demonstrated that Sjp40 could promote HSCs senescence via a p53/p21 dependent mechanism^21^. Although cellular senescence usually depends on p53 pathway or p16, increasing evidence suggests that cellular senescence could also be mediated by other manner. SKP2/P27 pathway is known to be involved in regulating cellular aging^33^. SKP2, an important ubiquitin E3 ligase complex, has been considered to be involved in multiple cellular processes including senescence, cell cycle, and apoptosis^34^. In addition, cyclin inhibitor P27 is normally viewed as a critical downstream target of SKP2, and there is a negative correlation between P27 and SKP2 expression. Once aging occurred, P27 could be upregulated and accompanied by SKP2 down-regulation. As S-J Kim *et al*., reported, ablation of galectin could down-regulate SKP2 expression and induce P27-dependent premature senescence that is reversed by SKP2 overexpression^35^. On the contrary, overexpression of SKP2 frequently displays a pro-oncogenic role and promotes cancer cell growth^36, 37^. Relevant studies have revealed that P27 is also associated with liver fibrosis. The activation of P27 could induce HSCs cell cycle arrest, leading to the inhibition of HSCs growth and the reduction of expression levels of pro-fibrogenic genes^38^. In present studies, our data also shows that P27 is obviously up-regulated in Sjp40-treated group and over-expression of SKP2 could down-regulate P27 level. Of note, over-expression of SKP2 or silencing of P27 could partially reverse the Sjp40-induced senescence in LX-2 cells. All together, SKP2/P27 pathway is implicated in the HSCs senescence induced by Sjp40.

Retinoblastoma (Rb), the tumor suppressor gene, is inactivated in various kinds of tumors, and it is usually involved in tumor progression via regulating cell differentiation, apoptosis, senescence and the cell cycle^39, 40^. Rb could stop the progression of G_1_-S phase transition and lead to G_1_ phase cell cycle arrest via binding to transcription factors of E2F family. Nevertheless, P-Rb has been regarded as the vital step in the progression of G_1_-S phase transition. Due to the inhibitory role of phosphorylation of Rb (P-Rb) on binding to E2F, the decrease of P-Rb induces G_1_ phase arrest^41^. As Hahm ER *et al*. reported, Honokiol promotes G_0_-G_1_ phase cell cycle block in association with suppression of P-Rb protein level in human prostate cancer cells^42^. In our experiment, we observed that the decreased level of P-Rb induced by Sjp40 was via SKP2/P27 pathway, which is consistent with PD-treated fibroblasts^23^.

Taken together, our current findings demonstrated that Sjp40 could obviously inhibit LX-2 cells activation and promote cell senescence *in vitro* experiment. Further studies suggested that Sjp40-induced senescence could be mediated by activating SKP2/P27 signaling in LX-2 cells, which provided novel insights into the mechanisms of treatment of liver fibrosis in the future.

## Methods

### Production and purification of Sjp40

According to the instructions, the recombinant Sjp40 protein was expressed and purified by the Ni-NTA His·Bind Resin (Novagen, USA), and identified by Western blot. The polymyxin B-agarose beads were used to remove the endotoxin of Sjp40 recombinant protein following our previous protocol^19^ and Sjp40 was eventually dissolved in PBS.

### Reagents

Primary antibodies for α-SMA, Rb, SKP2, P27 were purchased from Santa Cruz Biotechnology (USA). Primary antibodies for Caspase3, P-Rb, P-ERK were purchased from Cell Signaling Technology (USA). All of the secondary antibodies were purchased from Santa Cruz Biotechnology (USA). Recombinant human TGF-β1 and staurosporine (STS), a positive apoptosis stimulus, were obtained from Sigma (USA).

### Cell culture

Human hepatic stellate cell line, LX-2, was obtained from Xiang Ya Central Experiment Laboratory (China) and maintained in Dulbecco’s Modified Eagle Medium (DMEM, Gibco, USA) with 10% fetal bovine serum (FBS, Invitrogen, USA). Cells were cultured in a humidified incubator at 37 °C with 5% CO_2_ and stimulated with the additional Sjp40 (20 μg/mL) in complete media or media only control.

### Western Blot

The total proteins were extracted from LX-2 cells and protein concentration was quantified by Bradford method (Sangon, China). Protein samples were separated by SDS-PAGE (8–12%), transferred onto PVDF membranes (Merck, Germany), and blocked with 5% nonfat dry milk. Membranes were incubated with primary antibodies against P-Rb, P-ERK, and Caspase 3 (antibody dilution is 1:2000, Cell Signaling Technology, USA). P27, SKP2, Rb and α-SMA were obtained from Santa Cruz Biotechnology (1:200 dilution, USA). And then incubated with an appropriate second antibody which is a peroxidase-labeled anti-rabbit (1:5000 dilution, Abcam, USA) or anti-mouse (1:10000 dilution, Abcam, USA) immunoglobulin at room temperature. The target proteins were detected using a Schemiluminescence (ECL) kit (Merck, Germany). Bands were normalized with GAPDH and protein expressions were quantified by Image J.

### RNA interference

Cells were seeded into 6-well plates for 24 h. siRNA duplexes against P27(5′-AAGTACGAGTGGCAAGAGGTG-3′)^33^ and control duplex (5′-CCUACGCCACCAAUUUCGU-3′) were purchased from GenePharma (Shanghai, China). To knockdown P27 expression, we transfected siRNA-P27 (Si-P27, Genechem, China) or siRNA-control (Si-con, Genechem, China) in LX-2 cells. Then cells were treated with or without the Sjp40 (20 μg/mL) after transfection for 24 h.

### Special gene over-expression

pcDNA3.1 plasmid were digested with EcoRI and BamHI (TaKaRa, China), and CDS region of SKP2 (GenBank: NM_005983) was subcloned into pcDNA3.1 vector to generate the recombinant vector pcDNA3.1-SKP2. LX-2 cells were seeded into 6-well plates for 24 h and transfected with a human recombinant SKP2 expression vector (pcDNA3.1-SKP2) or with the corresponding empty vector (pcDNA3.1), using Lipofectamine 2000 (Invitrogen), and then were treated with or without stimulus for 24 h.

### SA-β-Gal assay

LX-2 cells were plated into 6-well plates for 24 h before the additional stimulus. Cells were stained using a senescence-associated β-galactosidase staining kit (Genmed, USA) according to the manufacturer’s instructions.

### Immunofluorescence assay

LX-2 cells were seeded in 6-well plates overnight before the additional stimulus. The cells were fixed with 4% paraformaldehyde for 1 h and permeabilized with 0.1% Triton-100 for 5 min. After blocking in 5% BSA for 2 h at room temperature, cells were incubated with anti-α-SMA antibody at 4 °C overnight, and then incubated with a fluorescence-labeled second antibody for 1.5 h at 37 °C. The cellular nuclei were also stained with Hoechst 33342 for 20 min and cells were photographed using fluorescence microscope.

### Statistical analysis

Date analysis was generated using one-way ANOVA or the independent samples T-test and all date were expressed as the mean ± SEM of three or four independent trials to determine the significant differences. *p* < 0.05 was considered significant.

## Electronic supplementary material

Supplement
