# Supplementary material for: Egg antigen p40 of Schistosoma japonicum promotes senescence in activated hepatic stellate cells via SKP2/P27 signaling pathway
Source: Sci Rep. 2017 Mar 21;7:275. doi: 10.1038/s41598-017-00326-1 (PMC5428252; doi:10.1038/s41598-017-00326-1)
Supplement: Supplementary file 1 — Supplement [file 41598_2017_326_MOESM1_ESM.doc]

**Egg antigen p40 of *Schistosoma japonicum* promotes senescence in activated hepatic stellate cells via SKP2/P27 signaling pathway**

Tianhua Xu, Jinling Chen, Dandan Zhu, Liuting Chen, Jianxin Wang, Xiaolei Sun, Bin Hu, Yinong Duan

# Figure S1

#
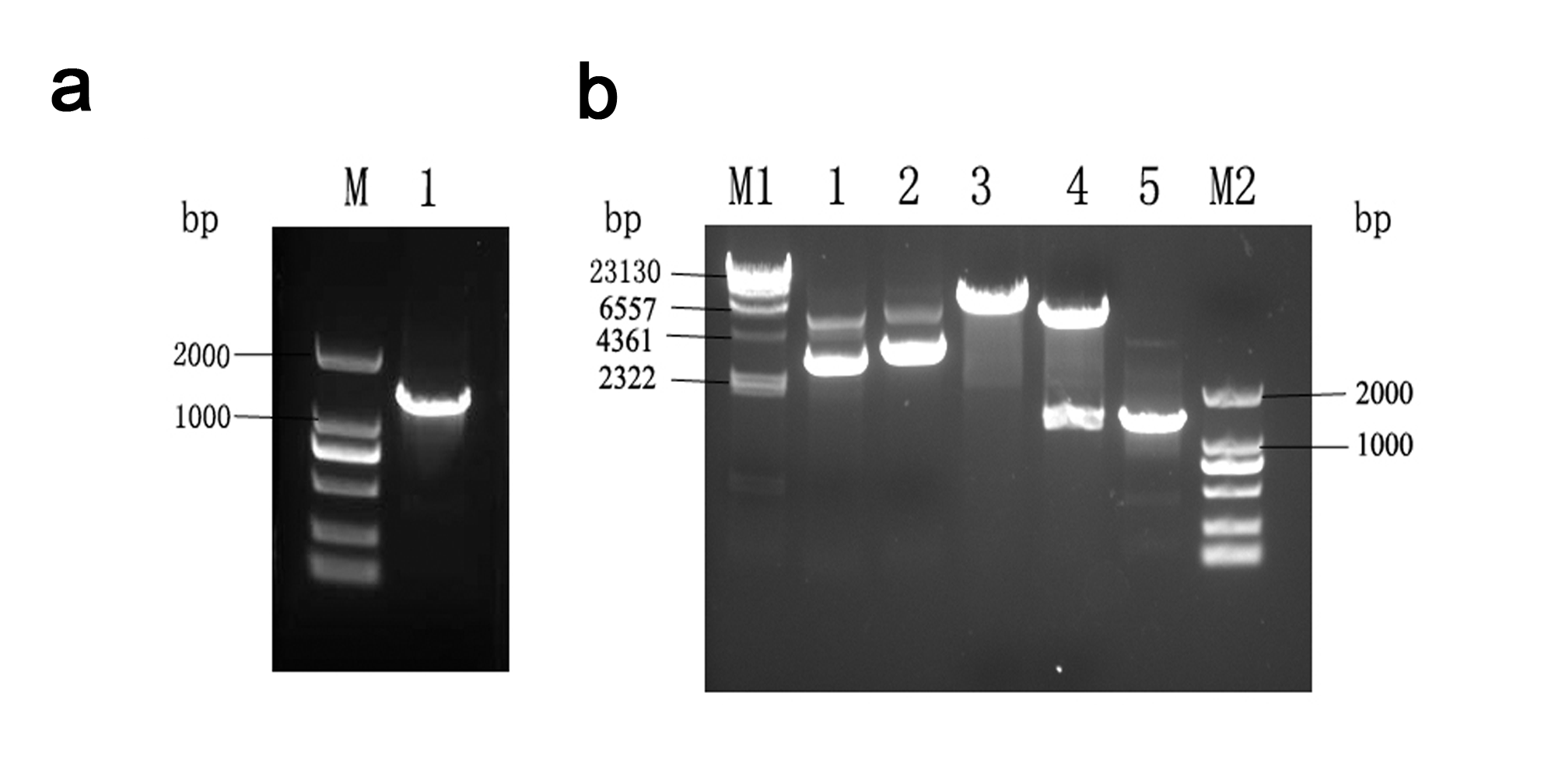


# Figure S1.The recombinant plasmids pcDNA3.1-SKP2 were constructed. (a) The amplification of SKP2 gene. M: DL2000 DNA Maker, 1: The aim gene fragment (1275bp); (b) Recombinant plasmid pcDNA3.1-SKP2 identified by restriction enzyme reaction. M1: λ-Hind III DNA Maker, 1: Empty vector pcDNA3.1, 2: Recombinant vector pcDNA3.1-SKP2, 3:  Mono-restriction endonuclease enzyme reaction, 4: Dual-restriction endonuclease enzyme reaction, 5: The amplification of SKP2 gene by PCR. M2: DL2000 DNA Maker.
